# Clinical Impact of CT-Based FFR in Everyday Cardiology: Bridging Computation and Decision-Making

**DOI:** 10.3390/biomedicines13081969

**Published:** 2025-08-13

**Authors:** Maria Bozika, Anastasios Apostolos, Kassiani-Maria Nastouli, Michail I. Papafaklis, Ioannis Skalidis, Dimitrios Terentes-Printzios, Antonios Karanasos, Christos Koutsogiannis-Korkontzelos, Georgios Boliaris, Spyridon Floropoulos, Anastasia Mavromati, Konstantinos Katsanos, Periklis Davlouros, Grigorios Tsigkas

**Affiliations:** 1 The Rongxiang Xu, MD, Center for Regenerative Therapeutics/Veves Lab, Beth Israel Deaconess Medical Center, Harvard-Affiliated Medical School, Boston, MA 02215, USA; mariabozika29@gmail.com; 2 Department of Medicine, Division of Cardiology, University Hospital of Patras, 26504 Patras, Greece; kassienmarie@gmail.com (K.-M.N.); m.papafaklis@yahoo.com (M.I.P.); akaranasos@hotmail.com (A.K.); kounikzx@gmail.com (C.K.-K.); gmpoliaris@gmail.com (G.B.); sfloropoylos@gmail.com (S.F.); natmavr@yahoo.gr (A.M.); pdav@upatras.gr (P.D.); gregtsig@upatras.gr (G.T.); 3 Department of Cardiology, Harefield Hospital, Royal Brompton and Harefield Hospitals, Guy’s and St Thomas’ NHS Foundation Trust, London UB9 6JH, UK; 4 First Department of Cardiology, Hippocration General Hospital, National and Kapodistrian University of Athens, 11527 Athens, Greece; dimitristerentes@yahoo.gr; 5 Institut Cardiovasculaire Paris-Sud, Hôpital Jacques Cartier, Ramsay-Santé, 91300 Massy, France; 6 Department of Interventional Radiology, School of Medicine, University of Patras, 26222 Patras, Greece; katsanos@upatras.gr

**Keywords:** FFR_CT_, coronary computed tomography angiography, coronary artery disease, non-invasive cardiac imaging, computational fluid dynamics, machine learning, artificial intelligence

## Abstract

A revolutionary non-invasive method for the thorough evaluation of coronary artery disease (CAD) is fractional flow reserve (FFR) obtained from coronary computed tomography angiography (CCTA). Computed tomography-derived FFR (FFR_CT_) assesses both the anatomical and functional significance of coronary lesions simultaneously by utilizing sophisticated computational models, including computational fluid dynamics, machine learning (ML), and Artificial Intelligence (AI) methods. The technological development, validation research, clinical uses, and real-world constraints of FFR_CT_ are compiled in this review. Large multicenter trials and registries consistently show that FFR_CT_ is a reliable gatekeeper to invasive coronary angiography (ICA) and increases diagnostic accuracy significantly when compared to coronary Computed Tomography Angiography (CTA) alone, especially in patients with intermediate-risk anatomy. Additionally, FFR_CT_ has demonstrated benefits in populations with in-stent restenosis (ISR) and in virtual procedural planning. Notwithstanding its advantages, the technique still requires high-quality imaging, and its practical application is constrained by expenses, processing requirements, and image distortions. Continuous developments in automation and deep learning should improve accessibility, effectiveness, and workflow integration in clinical settings. FFR_CT_ is expected to become more and more important in the individualized treatment of CAD by minimizing unnecessary invasive procedures and improving patient selection for revascularization.

## 1. Introduction

Invasive coronary angiography (ICA) is the gold standard for diagnosing coronary artery disease (CAD), offering direct visualization of luminal stenosis [1]. However, reliance on angiographic assessment alone is increasingly being challenged due to its limited capacity to predict the physiologic significance of stenoses and its high interobserver variability [2,3,4]. Even with the addition of quantitative coronary analysis, ICA does not always detect lesions that cause ischemia [5]. The FAME (Fractional Flow Reserve versus Angiography for Guiding Percutaneous Coronary Intervention) [5] and COURAGE (Clinical Outcomes Utilizing Revascularization and Aggressive Drug Evaluation) trials [6] established that patients with CAD causing significant flow limitation benefit from coronary revascularization. In contrast, individuals without evidence of ischemia gain minimal, if any, benefit from such procedures and may even be harmed. Moreover, the DEFER (Deferral Versus Performance of Percutaneous Coronary Intervention of Non-Ischemia-Producing Stenoses) trial [7] demonstrated that patients with coronary lesions not associated with flow limitation can be safely managed with optimal medical therapy (OMT) alone, achieving annual rates of myocardial infarction (MI) and mortality below 1%. Therefore, determining the hemodynamic significance of coronary stenoses during ICA with fractional flow reserve (FFR) is now recognized as a key tactic, allowing for both functional and anatomical assessment to guide lesion-specific therapy choices.

Invasive FFR provides an accurate evaluation of flow-limiting stenoses and is achieved by passing a pressure-sensor wire over the lesion during pharmacologically induced hyperemia. Deferral is preferred for lesions over this threshold, but values ≤0.80 are generally recognized to show functional relevance and predict better outcomes after revascularization [8]. Nevertheless, the invasiveness, expense, and procedural complexity of FFR prevent it from being widely used, which makes accurate non-invasive substitutes that can mimic its clinical utility necessary [9]. Anatomical and functional information is traded off in a number of non-invasive techniques, including stress echocardiography, myocardial perfusion imaging (MPI), stress cardiac magnetic resonance imaging (MRI), and coronary computed tomography angiography (CCTA) [10,11]. While MPI only produces segmental perfusion maps and has attenuation artifacts, stress cardiac MRI is still resource-intensive and lacks comprehensive vascular architecture, and stress echocardiography may not be able to detect small regional wall motion anomalies [12]. Although CCTA has a high negative predictive value and a good non-invasive assessment of coronary artery disease, its diagnostic accuracy is diminished in patients who have many stents, extensive calcifications, or arrhythmias [13,14].

Nonetheless, there has been momentum behind attempts to expand the use of CCTA beyond diagnosis to include procedural guidance. This is especially important for anatomically complex groups, including those who have had coronary artery bypass grafting (CABG) in the past. The GREECE (Computed tomoGRaphy guidEd invasivE Coronary angiography in patiEnts) trial with a previous coronary artery bypass graft surgery, a multicenter randomized study conducted across nine tertiary centers in Greece, demonstrated that CCTA-guided ICA in post-CABG patients significantly reduced fluoroscopy time and total procedure time, at the cost of higher total contrast volume and radiation dose compared to ICA alone [15,16]. Additionally, the BYPASS-CTCA (Computed Tomography Cardiac Angiography Before Invasive Coronary Angiography in Patients With Previous Bypass Surgery) trial, a large single-center study, confirmed that a CCTA-first approach reduces procedure time and, importantly, reduces contrast-induced nephropathy in CABG patients undergoing subsequent ICA [17]. Although these data do not address the physiologic impact of disease, they support the clinical use of high-resolution anatomical imaging, especially in complex coronary anatomies.

To overcome this limitation, computed tomography-derived fractional flow reserve (FFR_CT_) has become a revolutionary post-processing technique that enhances CCTA by using computational modeling to non-invasively assess lesion-specific ischemia. Based on patient-specific 3D anatomical reconstructions, the fundamental method simulates hyperemic coronary flow using computational fluid dynamics (CFD) and Navier-Stokes equation solutions [18,19]. To determine pressure and flow along the coronary tree, these models use estimates of microcirculatory resistance, aortic inflow, and geometric features. Similar to invasive FFR, hemodynamic significance is indicated by a threshold of ≤0.80. Recent developments, such as reduced-order solvers and machine learning techniques, have made it possible to conduct computations in real-time or almost real-time on conventional hardware without sacrificing accuracy, whereas early CFD-based solutions required several hours of off-site core-lab processing [20,21,22,23,24].

These advancements are indicative of a continuous change in cardiovascular imaging, where the combination of physiological understanding and anatomical precision is increasingly crucial for the best possible patient care. Leading this movement is FFR_CT_, which provides a clinically verified, non-invasive, and affordable substitute for conventional wire-based physiology. The aim of our review is to assess the existing FFR_CT_ platform’s clinical performance, validation data, regulatory status, and computational basis in the sections that follow. The scope of the literature considered encompasses prospective and retrospective validation studies, landmark randomized and observational trials, comparative evaluations with invasive and non-invasive reference standards, and cost-effectiveness analyses. Study selection prioritized methodological rigor, clinical applicability, and influence on practice, with emphasis on high-impact and recent evidence rather than exhaustive coverage of all published studies.

## 2. Clinical Indications for CCTA: Guidelines-Based Recommendations

The 2021 AHA/ACC/ASE/Chest/SAEM/SCCT/SCMR Multisociety Guidelines for the Evaluation and Diagnosis of Chest Pain and the 2021 Expert Consensus Document from the Society of Cardiovascular Computed Tomography (SCCT) provide detailed technical and clinical guidance for the use of CCTA in daily practice [25,26]. The recommendations are summarized in Table 1.

Most recently, a 2025 expert opinion from a joint SCAI/SCCT roundtable has further evaluated the expanding role of CCTA, not only for diagnostic assessment but also for procedural planning and guidance of percutaneous coronary intervention (PCI) [27]. Similar to its well-established role in other structural heart procedures, this consensus emphasizes the importance of CCTA in pre-procedural patient triage, complexity assessment, and shared decision-making. Additionally, they acknowledge the promise of sophisticated CCTA-based techniques, including vessel-specific myocardial mass quantification, virtual PCI planning, and FFR_CT_, to guide revascularization strategy in specific patient subgroups. The continuous development of clinical practice toward a more integrated, image-guided approach to the treatment of coronary artery disease is reflected in these perspectives.

## 3. An Overview of FFR_CT_ Techniques, Including Their Diagnostic Accuracy and Findings from Major Validation Studies

### 3.1. FFR_CT_ Based on the Heartflow Software

In the past decade, the use of FFR_CT_, combined with advanced computational analysis, has significantly improved the non-invasive assessment of coronary lesions, leading to better clinical outcomes. HeartFlow FFR_CT_ was the first method to non-invasively derive FFR from computed tomography angiography (CTA) data. Its computation requires the use of standard CCTA datasets, from which three-dimensional models of the entire coronary arterial tree and the ventricular myocardium are reconstructed with proprietary methods. Blood is considered a Newtonian fluid, and coronary flow and pressure fields are computed by solving the Navier–Stokes equations under appropriate boundary conditions using the finite element method on a parallel supercomputer. Flow and pressure are not known beforehand; thus, lumped parameter models of the coronary microcirculation are coupled to the outflow boundaries of the 3D coronary model. The computation is based on three key physiologic assumptions: (1) resting coronary flow matches myocardial demand, allowing the estimation of baseline flow relative to ventricular mass; (2) microvascular resistance at rest is inversely, though not linearly, related to vessel size; and (3) microcirculatory behavior during hyperemia is predictable [23]. The CFD-based FFR_CT_, generated by Heartflow, received Food and Drug Administration (FDA) approval in 2022.

The DISCOVER-FLOW (The Diagnosis of Ischemia-Causing Stenoses Obtained Via Non-invasive Fractional Flow Reserve) was the first trial in a series that validated HeartFlow FFR_CT_ and prospectively enrolled 103 patients and evaluated 159 coronary vessels [23]. HeartFlow FFR_CT_ demonstrated significantly higher diagnostic accuracy for identifying ischemia-causing lesions compared to CCTA stenosis alone, using invasive FFR ≤0.80 as the reference. Each FFR_CT_ analysis required approximately 5 h to complete, using high-performance computing resources. The DeFACTO (Determination of Fractional Flow Reserve by Anatomic Computed Tomographic Angiography) study utilized the second-generation FFR_CT_ algorithm (version 1.2) and evaluated a total of 252 patients and 407 coronary vessels with suspected or known CAD [28]. Expanded on these trials, the HTNXT (Analysis of Coronary Blood Flow Using CT Angiography) study conducted at 254 patients and 484 vessels with suspected CAD and found that FFR_CT_ demonstrated superior diagnostic performance over coronary CTA alone, particularly through improved specificity [29]. These results are due to the focused attention given to ensuring high-quality CT acquisitions, which involved the routine use of nitroglycerine and β-blockers, as well as imaging adjustments based on patient body size, and continuous improvements to the FFR_CT_ algorithm. In both the study by Yang et al. [30] and the PACIFIC trial substudy [31], FFR_CT_ demonstrated superior diagnostic performance compared to CCTA, while in the PACIFIC substudy, FFR_CT_ also outperformed SPECT and PET for detecting lesion-specific ischemia.

### 3.2. FFR_CT_ Based on the Siemens Healthcare Software

In a retrospective analysis, Renker et al. investigated the performance of a novel prototype algorithm for computing FFR from CCTA data, cFFR, on 53 patients with 67 coronary lesions, enabling time-efficient in-hospital calculation without the need for data transfer [32]. Patients with CCTA, ICA, and invasive FFR within 3 months were included. In this algorithm, an experienced observer, blinded to the invasive results, accepted or corrected the luminal contour and centerline proposals generated by the research software to produce a patient-specific 3D mesh of the coronary artery tree. The cFFR algorithm then simulated coronary blood flow using CFD, incorporating patient-specific boundary conditions (systolic and diastolic blood pressure, heart rate, left ventricular mass) to reflect hyperemic conditions via lumped cardiac and microcirculatory models. Flow computation was achieved by coupling reduced- and full-order models. On a per-lesion basis, CT-based FFR demonstrated an AUC of 0.92, with a sensitivity of 85%, specificity of 85%, and a positive predictive value of 71%, and a negative predictive value of 93% for identifying hemodynamically significant stenoses. Mean total time was 37.5 ± 13.8 min. Additionally, recent studies by Coenen et al. [21] and De Geer et al. [33] have demonstrated that cFFR, calculated on an on-site workstation, provides good diagnostic accuracy and correlates well with invasive FFR measurements.

The second-generation cFFR_ML_ (machine-learning approach for computation of FFR from coronary computed tomography), presented by Itu et al. in 2016, follows a two-phase framework [22]. In the offline phase, a training set of 12,000 synthetically generated coronary anatomies was paired with target cFFR values computed using a validated reduced-order CFD model. A fully connected deep neural network with four hidden layers was trained to learn the relationships between geometric features, extracted locally as well as from upstream and downstream vessel segments, and the corresponding CFD-derived cFFR values. In the online phase, a patient-specific three-dimensional anatomical model was reconstructed from CCTA data, and a predefined set of quantitative features was automatically extracted at each location along the vessel. These features were processed by the trained model to compute cFFR, which was visualized as a color-coded map of the coronary tree. Importantly, this method enabled near real-time computation, with an average execution time of ~2.4 ± 0.44 s [22].

Another method of FFR_CT_ presented by Toshiba Medical Systems combines structural deformation analysis and fluid dynamics to assess the functional significance of coronary stenoses. CCTA data acquired at four phases of the cardiac cycle (70%, 80%, 90%, and 99% of the R–R interval) are used to reconstruct three-dimensional models of the coronary tree and aorta, from which vessel deformation parameters are extracted. Boundary conditions are estimated using hierarchical Bayesian modeling, incorporating local luminal deformation, vessel compliance, and aortic volume change. Blood is represented as a non-Newtonian fluid, and coronary pressure and flow are calculated using a reduced-order one-dimensional fluid model based on the continuity, momentum, and constitutive equations. In the validation cohort, FFR_CT_ demonstrated good diagnostic accuracy and feasibility for point-of-care use, with a mean per-patient analysis time of 27.07 ± 7.54 min [34].

### 3.3. DeepVessel FFR Based on the Keya Medical Software

The computational load of conventional CFD-based FFR_CT_ restricted real-time clinical integration; hence, deep learning-based methods were developed to speed up prediction while preserving diagnostic precision. A fully Artificial Intelligence (AI)—driven approach to non-invasive functional evaluation was first clinically validated in 2019 when Wang et al. revealed DeepVessel FFR (DVFFR), one of the first such systems [35]. The first clinical validation of the DVFFR platform was conducted in a prospective, single-center study, involving 63 patients and 71 coronary vessels. In order to predict lesion-specific pressure gradients from CCTA data, the study presented a unique deep learning architecture (DBL-RNN). In the first stage, features such as lesion characteristics and proximal/distal markers are extracted from fully reconstructed three-dimensional arterial models using a multilevel neural network (MLNN) with three fully connected layers. These features are then processed in the second stage by a bidirectional recursive neural network (BRNN), which integrates spatial information from both proximal and distal vessel segments. The network was trained offline using ground truths generated by solving the Navier–Stokes equations, with invasive FFR as the reference standard, and optimized via stochastic gradient descent. For clinical application, raw CCTA DICOM images are uploaded directly to the platform, which performs the entire computation, without the need for manual segmentation, on a remote server. The computational time was 120 ± 13 s per case. These groundbreaking findings eased the path for more widespread validation. The ADAPT (Assessment of the Diagnostic Performance of DVFFR in Suspected Coronary Artery Disease) study was a multinational, multicenter, observational validation trial designed to evaluate the performance of DVFFR [36]. A total of 269 patients and 358 vessels with 30–90% coronary stenosis by CTA were enrolled across 10 clinical sites (5 in the U.S. and 5 in the EU), and results were compared against invasive FFR measurements as the reference standard. Currently, DVFFR has received regulatory approval in Europe, China, the US, and Singapore, as evidenced by its CE-marked (2018), NMPA-approved (2020), FDA-cleared (2022), and HSA-certified (2023) status [37].

### 3.4. uFFR_CT_ Based on the United Imaging Healthcare Software

Further advancements in FFR_CT_ techniques have focused on enhancing automation, reducing processing time, and improving accuracy in challenging scenarios such as calcified lesions or those within the diagnostic “gray zone” [38]. United-Imaging Healthcare’s uFFR_CT_ (computational fluid dynamics-based CT FFR) applies a three-dimensional CFD model to simulate coronary physiology using routine CCTA data. Anatomical models of the aorta and coronary tree are reconstructed via convolutional neural networks. Transluminal attenuation gradient (TAG) measurements are automatically extracted along centerlines of each artery and used to define outlet boundary conditions. A steady-state simulation is performed using a finite-volume solver of the incompressible Navier–Stokes equations on a desktop workstation. Blood is modeled as a Newtonian fluid, and hyperemic flow is simulated by adjusting distal resistance and aortic pressure based on empirically derived coefficients. In the multicenter validation study of 338 patients and 422 vessels, uFFR_CT_ demonstrated high diagnostic accuracy across a broad range of lesion complexities, including intermediate stenoses and high calcium scores. The computational time is 11.0 ± 2.8 min [38].

### 3.5. esFFR Based on the CAscope Software

Another algorithm, the esFFR (CFD-based CT-derived FFR), developed by Escope Tech, Inc., employs a fully automated CFD framework using CCTA-derived models of the aortic root and coronary arteries, reconstructed via a deep learning–enhanced level-set segmentation pipeline [39]. Vessel centerlines are extracted by a connectivity-preserving 3D convolutional neural network trained on over 500 annotated datasets, ensuring complete coronary tree modeling. Hyperemic boundary conditions are determined by estimating flow velocity and regional myocardial blood flow using a second CNN trained on CCTA-phase pairs, with validation against a standardized CT perfusion database. Blood flow is simulated using a GPU-accelerated immersed boundary CFD solver based on the incompressible Navier–Stokes equations, and the average time for complete analysis is 4.6 ± 1.3 min on a personal computer.

All diagnostic performance results from the studies described above are presented in Table 2.

## 4. FFR_CT_ in Clinical Applications

### 4.1. Stable CAD

The application of FFR_CT_ in patients with stable CAD represents a transformative advance in non-invasive cardiac functional assessment. The diagnostic performance of FFR_CT_ has been validated against invasive FFR across multiple studies, while recent real-world data and meta-analyses have begun to delineate the field surrounding clinical and prognostic implications.

The earliest systematic attempt to consolidate the diagnostic accuracy of FFR_CT_ was published in 2017, in a vessel-level based meta-analysis of 908 lesions from 536 patients [40]. The investigators found that, overall, the per-vessel accuracy reached 81.9% (95% CI, 79.4–84.4%), but with clear heterogeneity depending on the FFR_CT_ value itself. At the high (>0.90) and low (≤0.60) ends of the spectrum, agreement with invasive FFR was strong—97.9% and 86.4%, respectively. However, diagnostic concordance in the “grey zone” (FFR_CT_ 0.70–0.80) was only 46.1%. Using logistic regression, the authors established interpretive thresholds; values exceeding 0.83 or falling below 0.63 attained over 82% diagnostic accuracy; stricter thresholds (e.g., >0.93 or <0.53) were necessary to achieve 95% certainty. This detailed stratification illustrates the necessity for clinical prudence in the management of patients exhibiting borderline FFR_CT_ results, particularly in cases of intermediate coronary stenoses, which were inadequately represented in this dataset (solely 12.8% of lesions were classified within invasive FFR 0.70–0.80). This study confirms that FFR_CT_ is not uniform across the full spectrum of disease presentation, necessitating careful contextual interpretation for borderline values.

A substantial body of evidence, including both prospective and multicenter studies, now establishes that FFR_CT,_ particularly with deep learning and machine learning-based algorithms, offers significant clinical value beyond anatomic imaging alone. The multicenter MACHINE (Machine Learning Based CT Angiography Derived FFR: A Multi-Center Registry) consortium study, for example, demonstrated that CT-FFR_ML_ significantly enhanced diagnostic accuracy compared to visual CTA, particularly through its ability to reclassify false-positive findings [41]. Among 85 false-positive CTA interpretations, 62 (73%) were correctly reclassified as non-ischemic by CT-FFR_ML_, thereby indicating its clinical utility in reducing unnecessary invasive coronary angiographies. This study also addressed image quality as an important factor in diagnostic performance. Specifically, patients in the highest image quality tertile achieved an accuracy of 91%, compared to 75% in the lowest tertile. The authors further emphasized that even though the CT-FFR_ML_ model provided computational efficiency, the diagnostic accuracy of FFR_CT_ methods decreased as invasive FFR values got closer to the 0.80 threshold. This is an important point to make because 57% of the lesions studied were physiologically intermediate (FFR 0.70–0.90). These observations were confirmed by von Knebel Doeberitz et al., who assessed the diagnostic utility of FFR_CT_ derived from deep learning, in combination with quantitative plaque features extracted from coronary CTA [42]. The investigators found that individual plaque characteristics such as lesion length (OR 1.15, *p* = 0.037), non-calcified plaque volume (OR 1.02, *p* = 0.007), and the presence of napkin-ring sign (OR 5.97, *p* = 0.014) were independently associated with ischemia, although with only modest discrimination when considered alone. When plaque characteristics were combined with conventional stenosis grading ≥ 50%, the predictive AUC improved to 0.83. Specifically, CT-FFR_ML_ ≤ 0.80 achieved a sensitivity of 82%, specificity of 94%, positive predictive value of 88%, and negative predictive value of 92%. Thus, this enhanced discriminatory power reinforces the role of CT-FFR_ML_ in the mitigation of overdiagnosis and reducing unnecessary ICA referrals by filtering out morphologically severe but functionally irrelevant lesions. This improves cost-effectiveness and reduces patient exposure to unnecessary procedures.

The recent surge in real-world and pragmatic trial data has further substantiated the clinical utility of FFR_CT_-based strategies. PLATFORM (The Prospective Longitudinal Trial of FFR_CT_) study further assessed the impact of FFR_CT_-guided diagnostic strategies on clinical decision-making and outcomes in patients with suspected stable CAD [43]. Among 380 patients with planned ICA, the incorporation of FFR_CT_ into diagnostic workflows resulted in a substantial reduction in the proportion of patients undergoing ICA without obstructive CAD. Specifically, from 73.3% in the usual care group to just 12.4% in the FFR_CT_ arm (risk difference 60.8%, 95% CI: 53.0–68.7; *p* < 0.0001); 61% of ICAs were cancelled after FFR_CT_ analysis. Despite the reduction in invasive procedures, the 90-day incidence of MACE remained low and comparable between groups. Furthermore, functional information (FFR or FFR_CT_) was available in 97.4% of the FFR_CT_-guided ICA group, compared to only 44.4% in the usual care cohort (*p* < 0.0001), ensuring that most revascularizations were guided by objective physiological assessment. This double advantage, namely, increasing triage effectiveness without sacrificing safety, establishes FFR_CT_ as a clinically useful gatekeeper to ICA in the assessment of stable chest pain. After 1 year, both the conventional and CTA/FFR_CT_ care groups exhibited an equal number of major adverse cardiac events. The trial’s 1-year outcome data indicated that the care guided by CCTA/FFR_CT_ resulted in lower costs while maintaining equivalent quality of life and clinical outcomes [44].

The FORECAST (Fractional flow reserve derived from computed tomography coronary angiography in the assessment and management of stable chest pain) trial and subsequent contemporary randomized studies moved the field closer to current practice standards. This trial was a multicenter randomized study designed to evaluate whether a diagnostic strategy incorporating CCTA with selective FFR_CT_ could improve resource utilization, clinical outcomes, and cost-efficiency in patients presenting with stable chest pain [45]. A total of 1400 patients were randomized to either standard care or a CCTA-first approach with selective FFR_CT_ application for lesions with >40% stenosis. Standard care in this study namely refers to a diagnostic pathway where patients with a high pre-test likelihood of significant coronary disease are referred directly for invasive coronary angiography, while those with intermediate pre-test likelihood are referred for non-invasive evaluation, which could include a range of stress testing modalities, such as stress echocardiography, stress cardiac magnetic resonance, nuclear medicine perfusion imaging, or exercise electrocardiography, as well as CCTA without FFR_CT_. In the standard care group, the initial test was pre-specified by the clinical team before randomization, and all subsequent management decisions were based on the results of this test and clinical judgment. The use of ICA was significantly reduced in the experimental arm (19% vs. 25%, *p* = 0.01), with a 52% reduction in procedures revealing unobstructed arteries. However, the primary endpoint of total cardiac cost at nine months did not differ significantly between groups (mean difference: +£114, *p* = 0.10). Clinical outcomes were also comparable, including major adverse cardiovascular and cerebrovascular events (MACCE) (10.2% vs. 10.6%) and changes in angina burden or quality of life. Although these results highlight the limited cost savings possible in health systems where CCTA is already the standard non-invasive modality, they also confirm the safety and triage benefit of FFR_CT_ in reducing unnecessary ICA, especially for patients with anatomically intermediate lesions. Parallel to these, single-center and multicenter real-world data have consistently underscored the efficiency gains and safety profile associated with FFR_CT_. Furthermore, in a prospective, single-center study, Rabbat et al. evaluated 387 patients with suspected stable CAD undergoing coronary CTA with selective FFR_CT_, compared to 44 control patients managed with CTA alone [46]. Selective FFR_CT_ use resulted in a significant reduction in ICA rates for those with obstructive CAD (45% vs. 80%), and a higher proportion of patients undergoing ICA were revascularized (62%). Critically, among patients with FFR_CT_ >0.80 who deferred ICA, there were no MACE over a mean follow-up of 440 days. FFR_CT_ was feasible in more than 90% of cases, and this study demonstrated the safety and effectiveness of combining CTA and selective FFR_CT_ to defer unnecessary ICA and improve downstream patient management.

Parallel to these, single-center and multicenter real-world data have consistently underscored the efficiency gains and safety profile associated with FFR_CT_. The IMPACT-FFR (Real world impact of added FFR-CT to coronary CT angiography on clinical decision-making and patient prognosis) study retrospectively evaluated the real-world consequences of integrating FFR_CT_ into the diagnostic algorithm for stable CAD by retrospectively comparing 136 patients assessed with FFR_CT_ to 224 evaluated with CCTA alone [47]. This study addressed this by showing that, without a corresponding rise in adverse events, FFR_CT_-guided therapy was linked to a significant decrease in ICA rates (19.9% vs. 51.3%, *p* < 0.001) and revascularization operations (13.2% vs. 24.6%, *p* = 0.01). The incidence of MACE during a 12-month period was quantitatively lower in the FFR_CT_ group (2.2% vs. 4.0%, *p* = 0.04), despite the decrease in downstream testing and revascularizations (13.2% vs. 24.6%, *p* = 0.01). Similarly, a prospective, single-center study included 1133 patients with suspected CAD and intermediate stenosis, comparing anatomical CCTA alone (n = 567) to CT-FFR_ML_ (n = 566) [48]. There were fewer ICA referrals (20.3% vs. 27.5%, *p* = 0.003) and fewer ICAs with non-obstructive findings (19.8% vs. 33.3%, *p* = 0.03) in the CT-FFR_ML_ group at 90 days. The CT-FFR_ML_ group had a considerably higher revascularization-to-ICA ratio (53.1% vs. 33.3%, *p* = 0.002). The CT-FFR_ML_ group also experienced MACE at 1 year (3.9% vs. 6.7%, HR: 1.73; *p* = 0.04). The authors emphasized that CT-FFR_ML_ strengthened prognostic stratification, reduced unnecessary ICAs, and improved ICA efficiency compared to anatomy alone. They also support its wider application as a “non-invasive gatekeeper” in stable CAD with intermediate lesions. Additionally, in the multicenter, randomized TARGET trial, Yang et al. assessed the clinical and economic impact of an on-site machine learning–based FFR_CT_ strategy on clinical decision-making in patients with stable coronary artery disease and intermediate stenoses [49]. Among 1216 participants, FFR_CT_-guided care significantly reduced the proportion of patients undergoing invasive coronary angiography without obstructive disease or without subsequent intervention within 90 days (28.3% vs. 46.2%, *p* < 0.001), compared to standard care guided by stress testing. This reduction was primarily driven by the improved identification of non-obstructive disease. Interestingly, the FFR_CT_ group experienced revascularization more frequently at 90 days (49.7% vs. 42.8%, *p* = 0.02), but this did not result in improvements in major cardiovascular events, quality of life, or symptoms after one year. In addition to demonstrating the therapeutic value of on-site CT-FFR_ML_ for procedural triage, this study also shows a trend toward cost savings that fell short of statistical significance (*p* = 0.07) and a neutral effect on patient-centered outcomes. Recent advances have also focused on point-of-care and on-site machine learning solutions. In a retrospective study, Giannopoulos et al. evaluated the diagnostic performance of a novel on-site deep learning–based FFR_CT_ algorithm in 74 lesions from 59 patients undergoing invasive coronary angiography with FFR measurement [50]. The algorithm, designed for real-time clinical application, achieved high diagnostic accuracy (AUC: 0.975, sensitivity of 93.5%, specificity of 97.7%) for identifying significant hemodynamic stenoses (FFR ≤ 0.80). Importantly, among the patients with severely calcified lesions, the diagnostic performance remained excellent with an AUC of 0.991, with a sensitivity of 94.7% and a specificity of 95%. The time required for mean analysis per patient was 7 min and 54 s. Additionally, Pan et al. conducted a large, observational study evaluating the impact of DVFFR on clinical management in 229 patients with suspected CAD, encompassing 485 coronary lesions [51]. DVFFR demonstrated excellent per-lesion diagnostic performance when compared to invasive coronary angiography, with an AUC of 0.86 (95% CI, 0.82–0.89), sensitivity of 67%, and specificity of 88%. Of particular note, this study emphasized that DVFFR was able to detect functional ischemia in a subset of patients with only mild to moderate stenosis on invasive angiography, with 14.6% of ICA-negative patients being reclassified as having functionally significant disease according to FFR_CT_. However, there were limitations as well. DVFFR showed reduced reliability in cases of small-caliber arteries (<2 mm), occluded vessels, or significant calcification, underscoring the necessity of carefully integrating it with anatomical and clinical data. Although DVFFR has developed into a rapid and precise method for routine risk assessment and non-invasive lesion evaluation, these results indicate the need for thorough consideration of lesion characteristics and technical limitations for the best possible patient management.

Finally, meta-analytic data confirmed the advantages of FFR_CT_, including five studies (3 randomized and two observational) with a total population of 5282 patients, and evaluated the effectiveness of FFR_CT_ as a first diagnostic approach in stable CAD [52]. The FFR_CT_ method was linked to significantly fewer invasive procedures than conventional, cardiovascular testing, especially in individuals without obstructive disease (OR for ICA without obstructive CAD: 6.63; 95% CI 4.79–9.16; *p* < 0.001). Significantly, revascularization referrals were more common among FFR_CT_ patients (OR: 0.48; 95% CI: 0.38–0.62; *p* < 0.001), indicating more accurate detection of functionally important lesions. Key 12-month clinical outcomes, such as MI, all-cause mortality, and unscheduled interventions, did not show any significant differences despite these management variations. These results support FFR_CT_’s ability to select patients who are unlikely to benefit from invasive testing while also allowing for prompt revascularization in suitable candidates, achieving a balance between clinical safety and diagnostic effectiveness.

### 4.2. Acute Chest Pain

A promising area in non-invasive cardiovascular diagnostics is the use of FFR_CT_ in the setting of acute chest pain. Although its usefulness in patients with intermediate coronary stenoses and stable symptoms is well established, its applicability in the acute setting and especially in patients who exhibit signs of myocardial injury is still being studied. For patients with mild to moderate stenosis detected by CCTA, the possibility of undergoing functional lesion assessment without the need for pharmacologic hyperemia is very intriguing. Nevertheless, there are currently little data to support the routine usage in acute presentations. The reliability of FFR_CT_ as a risk stratification tool for acute chest pain must be established through ongoing research in order to identify which patients can be safely released and which require escalation to invasive evaluation.

The initial application of computational hemodynamic assessment in the context of acute coronary syndrome was illustrated by the EMERALD (Exploring the Mechanism of Plaque Rupture in Acute Coronary Syndrome Using Coronary CT Angiography and Computational Fluid Dynamics) study, which investigated whether combining anatomical, plaque, and hemodynamic features derived from CTA could improve lesion-level prediction of ACS [53]. This study retrospectively analyzed 216 lesions (66 culprit and 150 non-culprit) in 72 patients who had undergone CTA 1 to 24 months before their ACS event. Each lesion was evaluated for both adverse plaque characteristics, such as low-attenuation plaque, positive remodeling, and napkin-ring sign, and non-invasive hemodynamic indices derived from computational fluid dynamics, including FFR_CT_, ΔFFR_CT_, wall shear stress (WSS), and axial plaque stress (APS). Culprit lesions showed greater diameter stenosis (DS) (55.5% vs. 43.1%, *p* < 0.001), higher rates of adverse plaque characteristics (APC) (80.3% vs. 42.0%, *p* < 0.001), lower FFR_CT_ (0.72 vs. 0.79, *p* = 0.006), larger ΔFFR_CT_ (0.17 vs. 0.06, *p* < 0.001), higher WSS (221.8 vs. 145.5 dyn/cm^2^, *p* < 0.001), and elevated axial plaque stress (APS) (2585.9 vs. 1734.7 dyn/cm^2^, *p* = 0.006). Adding adverse hemodynamic characteristics (AHC) to %DS, lesion length, and APC significantly improved predictive performance (c-index: 0.789 vs. 0.747; *p* = 0.014). Lesions with both APC and AHC had a higher risk of being the culprit (HR 11.75; 95% CI: 2.85–48.51; *p* = 0.001). Among all variables, ΔFFR_CT_ contributed the greatest individual information gain for lesion discrimination. Taking the integration of physiology and anatomy a step further, the EMERALD-II (Exploring the Mechanism of Plaque Rupture in Acute Coronary Syndrome Using Coronary Computed Tomography Angiography and Computational Fluid Dynamics II) study was a large, international, retrospective, multicenter analysis investigating whether AI-enabled quantitative coronary plaque and hemodynamic analysis (AI-QCPHA) could improve prediction of future culprit lesions in patients who subsequently experienced ACS [54]. In 351 patients with ACS and prior CTA, 363 culprit and 2088 non-culprit lesions were identified and split into derivation (n = 243) and validation (n = 108) cohorts. Adding five AI-derived features (ΔFFR_CT_, plaque burden, total plaque volume, low-attenuation plaque volume, and averaged percent myocardial blood flow) to the reference model (which consisted of the Coronary Artery Disease Reporting and Data System, a standardized classification for stenosis severity, and high-risk plaque, defined as lesions exhibiting two or more adverse plaque characteristics) significantly improved AUC for culprit lesion prediction, from 0.76 to 0.86 in the derivation set, and from 0.78 to 0.84 in the validation set (both *p* < 0.001). ΔFFR_CT_ was the strongest individual predictor (SHapley Additive explanation analysis); each 0.1 unit increase in ΔFFR_CT_ was associated with an OR of 1.83 (95% CI: 1.40–2.39; *p* < 0.001). Furthermore, in a substudy of the ROMICAT II trial, FFR_CT_ was applied to a cohort of emergency department patients with acute chest pain, aiming to evaluate its feasibility and clinical relevance in an acute care context [55]. Even though non-optimized CT scanners and pre-2011 imaging protocols were employed, FFR could be computed in 59% of the 68 patients. A strong association emerged between FFR_CT_ ≤ 0.80 and the presence of ACS (relative risk 4.03, 95% CI: 1.56–10.36). Particularly, 85% of those diagnosed with ACS had abnormal FFR_CT_, and such patients were more likely to undergo revascularization (37.5% vs. 10.7%). The analysis showed that FFR_CT_ added independent predictive value beyond anatomical stenosis, particularly in lesions with high-risk features such as napkin-ring sign, positive remodeling, and low attenuation.

Further emphasizing real-world integration, in a retrospective single-center study evaluating the implementation of FFR_CT_ in the emergency department, Fischer et al. demonstrated that a strategy incorporating CCTA followed by off-site FFR_CT_ analysis may help safely defer invasive testing in patients presenting with acute chest pain [56]; 25 out of the 59 patients were discharged from the Emergency Department and 17 out of the 25 had non-ischemic FFR_CT_ values (>0.80), and none of them had MACE at 30 days Importantly, all those with FFR_CT_ > 0.8 who underwent further stress testing or invasive angiography were free of functionally significant CAD, supporting the substantial negative predictive value of FFR_CT_ in this context. With a median turnaround time of 3.5 h and an estimated one-third of patients handled before findings were available, this study also brought attention to operational obstacles to real-time clinical integration. Expanding the evidence base, Chinnaiyan et al. retrospectively assessed the clinical utility of FFR_CT_ in the emergency department setting for patients presenting with acute chest pain [57]. Of 555 screened individuals, 297 underwent FFR_CT_ and CTA. The clinical performance of FFR_CT_ demonstrated reliability, where no myocardial infarctions or fatalities were noted at 90 days among patients with negative FFR_CT_, confirming the safety of postponing invasive testing in this cohort. Although the FFR_CT_ group experienced lower event rates (2.7% vs. 4.3%) compared to the standard CTA group, this difference did not reach statistical significance. Cost analysis revealed that patients with negative FFR_CT_ incurred significantly lower costs, and, on follow-up angiography, more than half with negative FFR_CT_ had no obstructive disease, further demonstrating the diagnostic value and potential for cost reduction. Distinctly, Gaur et al. investigated the diagnostic accuracy of FFR_CT_ for evaluating non-culprit lesions in patients with recent ST-segment elevation myocardial infarction (STEMI) [58]. One month following initial percutaneous coronary intervention, 124 non-culprit vessels on 60 patients were evaluated as part of the group. While FFRCT demonstrated moderate performance (accuracy 64%, sensitivity 93%, specificity 49%) and outperformed CTA alone, correlation with invasive FFR was only modest (Pearson r = 0.57), especially in vessels with low volume-to-mass ratio. Diagnostic accuracy rose to 83% in the upper tertile of vessel volume-to-mass ratio (≥65 mm^3^/g) but dropped to 56% in the lowest tertile (<49 mm^3^/g), highlighting the influence of post-infarction vascular and microvascular changes on FFR_CT_ reliability. These findings imply that the accuracy of non-invasive FFR assessment may be jeopardized by changed cardiac physiology and microvascular dysfunction after STEMI. Therefore, without considering anatomical-functional characteristics such as vascular size, FFR_CT_ may not be routinely suggested in the early post-STEMI period, even though it performs better than anatomical assessment alone [59].

### 4.3. Non-ST-Segment Elevation Myocardial Infarction (NSTEMI)

Significant diagnostic challenges still exist when evaluating individuals who present with non-ST-segment elevation acute coronary syndromes (NSTE-ACS). ECG results and clinical presentation help with early triage, but they frequently fall short of the level of accuracy needed for a conclusive diagnosis. Although moderate elevation of highly sensitive troponin (hs-Tn), usually up to three times the upper reference limit, often leads to diagnostic uncertainty, hs-Tn assays have improved the early detection of myocardial injury. Interestingly, ICA eventually reveals that up to half of high-risk NSTE-ACS patients with moderate hs-Tn increases do not have obstructive coronary artery disease [60]. The necessity for better non-invasive stratification is highlighted by this diagnostic inefficiency. Particularly in groups with low to intermediate risk, CCTA has shown strong efficacy in ruling out severe coronary disease. Regarding FFR_CT_, recent studies indicate that it may not only be feasible in this setting but also offer superior diagnostic accuracy compared to anatomical imaging alone [61,62,63]. Although its effectiveness in treating stable coronary artery disease and, to a lesser degree, patients with unstable angina, its role in assessing NSTE-ACS has been poorly understood. Recent studies, however, have begun to examine its viability and clinical significance in this urgent situation.

In a prospective multicenter study evaluating high-risk NSTE-ACS patients, Meier et al. assessed whether a coronary CTA-first strategy augmented by FFR_CT_ could safely exclude functionally significant stenoses and thereby reduce unnecessary ICA [64]. Of 168 enrolled patients, 151 (92%) had sufficient image quality for FFR_CT_ analysis. Among these, 66% were found to have significant lesions requiring revascularization. Among 168 enrolled patients, 92% had suitable image quality for FFRCT analysis, and 66% had significant lesions needing revascularization. CCTA alone showed high sensitivity (93%) and NPV (80%), with FFRCT modestly improving these to 94% and 85%, respectively, though not reaching statistical significance at the patient level. At the lesion level, FFR_CT_ offered significantly better diagnostic accuracy (AUC 0.84 vs. 0.64, *p* < 0.01), mainly due to improved specificity (81% vs. 70%), and could have prevented 22% of invasive angiograms without compromising safety. Moreover, Zimmerli et al. conducted a prespecified sub-analysis of a multicenter study to assess the diagnostic performance of FFR_CT_ in evaluating non-culprit lesions in high-risk NSTE-ACS patients [65]. Among 49 patients (67 lesions), FFR_CT_ demonstrated a sensitivity of 93%, specificity of 79%, PPV of 76%, NPV of 94%, and an overall accuracy of 85%, using invasive FFR as reference. Importantly, 31 of 33 non-significant lesions by FFR_CT_ were confirmed by invasive assessment. These findings emphasize that FFR_CT_ has the potential to safely defer invasive testing for intermediate non-culprit stenoses, streamline care, and reduce repeat angiography, though the study notes moderate PPV and the need for integrated clinical workflows as practical limitations. In parallel, prospective research headed by Poon (NCT03329469) is presently being conducted to evaluate invasive FFR and FFR_CT_ in patients with NSTEMI. The main goal is to evaluate FFR_CT_’s diagnostic performance in comparison to the invasive gold standard, considering factors like sensitivity, specificity, and predictive values. Secondary endpoints will look at measures related to healthcare utilization, such as overall cost of care and revisit rates. Achieving consistently high-quality CT imaging will be crucial for the successful application of such non-invasive procedures in the future, especially when sophisticated methods like quantitative plaque analysis are progressively included in clinical workflows.

### 4.4. Clinical Decision Making

Clinical decision-making in complex coronary artery disease increasingly relies on the integration of both anatomical and functional information. Recent evidence suggests that this approach may closely align with treatment recommendations based on invasive evaluation, especially in multivessel and left main disease [66]. The integration of FFR_CT_ into clinical decision-making has demonstrated the capacity to influence revascularization strategies in patients with complex multivessel coronary artery disease. Early on, in a real-world, single-center observational study, Nørgaard et al. assessed the impact of FFR_CT_ on decision-making in patients with suspected stable CAD [67]; 185 patients were referred for FFR_CT_, with 31% of patients and 10 of the vessels having FFR ≤ 0.80. Among those patients 29% underwent ICA, 37 patients underwent FFR or iFR. There was a correct correlation between FFR_CT_ ≤ 0.80 with FFR ≤ 0.80 or iFR ≤ 0.90 (in 73% and 70% of the patients, respectively). No adverse events were observed in the patient population with FFR_CT_ > 0.80. Thus, this study suggests that the implementation of FFR_CT_ in clinical practice may significantly impact downstream diagnostic strategies. Building on these insights, the ADVANCE (Assessing Diagnostic Value of Non-invasive FFR_CT_ in Coronary CarE) registry prospectively enrolled 5083 patients and assessed the 1-year impact of FFR_CT_ on clinical decision-making and outcomes in patients with suspected CAD [68]. Results demonstrated that revascularization was significantly more frequent in patients with an FFR_CT_ ≤ 0.80 (38.4%) compared to those with values > 0.80 (5.6%; RR: 6.87; 95% CI: 5.59–8.45; *p* < 0.001). Moreover, the incidence of cardiovascular death or MI at one year was significantly lower in patients with an FFR_CT_ > 0.80 compared to those with an FFR_CT_ ≤ 0.80 (0.2% vs. 0.8%; RR: 4.22; 95% CI: 1.28–13.95; *p* = 0.01). Finally, MACE rates were lower in patients with FFR_CT_ > 0.80 than in those with an FFR_CT_ ≤ 0.80 (0.6% vs. 1.2%), though without reaching statistical significance (*p* = 0.06). Parallel to the ADVANCE registry, the selective real-world use of FFR_CT_ for guiding ICA was addressed in a 2017 study involving 3974 patients undergoing CTA, of whom 375 underwent FFR_CT_. Revascularization was performed in 53% of the patients, and the rates were significantly higher among patients triaged via FFR_CT_ compared to conventional workup (44% vs. 21% for 30–69%, and 69% vs. 46% for 70–89%, *p* < 0.05). FFR_CT_ also achieved a high diagnostic yield, correctly identifying the treated vessel in 93% of patients referred to ICA. These results supported a tailored, physiology-guided approach to ICA and revascularization, rather than a one-size-fits-all anatomical triage. The incorporation of FFR_CT_ into heart team decision-making was explored in the SYNTAX III REVOLUTION substudy, which brought a unique perspective by examining how FFRCT could impact not only whether to revascularize, but also the overall revascularization strategy, namely, the choice between PCI and CABG [69]. Here, FFR_CT_ data changed heart team treatment decisions in 7% of patients and altered vessel-level plans in 12%. Integration of non-invasive physiology led to a 15.5% reclassification to a lower SYNTAX tertile, reducing the functional three-vessel disease rate from 92.3% to 78.8%, and providing a more individualized revascularization framework. This transition marked a shift from solely anatomical scoring toward functional disease assessment, showing the real clinical impact of FFR_CT_ in multidisciplinary meetings.

Expanding the potential of FFR_CT_, more recent studies have investigated its use in virtual procedural planning and as a predictor of post-PCI physiology. In a multicenter, investigator-initiated, prospective study, Sonck et al. evaluated a FFR_CT_ virtual planning platform designed to simulate post-PCI physiology and guide revascularization strategy [70]. This study enrolled 120 patients with 123 vessels, all of whom underwent PCI. Before PCI, patients underwent CTA, and additionally, Optical Coherence Tomography (OCT) was conducted before and after stent implantation. According to their outcomes, there was a mean difference of 0.00 between planner FFR (0.22 ± 12) and invasive FFR (0.22 ± 0.14), with limits of agreement ranging from −0.26 to 0.26. Regarding the Minimal Stent Area, the Planer had a 0.66 m^2^ mean difference in comparison to OCT (5.6 ± 2.0 mm^2^ vs. 5.0 ± 2.2 mm^2,^ respectively, with limits of agreement −1.7 to 3.0). The ability of FFR_CT_ Planer to predict FFR values remained high in focal and diffuse disease, as well as in high and low calcium burden, reinforcing the readiness of FFR_CT_-based virtual planning tools for routine clinical application in PCI optimization. Continuing this direction, Van Belle et al. evaluated the impact of an interactive FFR_CT_–based interventional planning tool on revascularization strategy among patients with at least one stenosis ≥ 40% [71]. In this prospective analysis of 101 patients (327 stenoses), treatment recommendations derived from ICA were compared against those obtained using a FFR_CT_. Use of the planner led to a change in management in 31% of lesions and altered the revascularization strategy in 45% of patients. Importantly, revascularization rates increased numerically (from 71.9% to 78.2%, *p* = 0.01) after planner integration. Among 78 ICA-targeted PCI segments, 22% were no longer recommended for intervention following physiological reassessment, and 22% required modification of stent length or positioning. Addressing long-term prognostic implications, a Danish multicenter substudy of the ADVANCE registry, involving 900 patients with new-onset stable angina, confirmed that abnormal FFR_CT_ ≤ 0.80 was associated with substantially higher three-year rates of all-cause death or nonfatal MI (6.6% vs. 2.1%; RR 3.1; *p* < 0.001) and cardiovascular death or MI (5.0% vs. 0.6%; RR 8.8; *p* = 0.001) [72]. The predictive value of FFR_CT_ remained significant even in patients with extensive calcification (calcification coronary artery score ≥ 400), with clear improvement in risk stratification over CTA alone (AUC 0.74 vs. 0.62, *p* < 0.001).

### 4.5. Transcatheter Aortic Valve Implantation (TAVI)

Since all candidates for transcatheter aortic valve implantation (TAVI) often undergo CT imaging for eligibility evaluation and procedure preparation, the use of FFR_CT_ is very attractive in this population [73,74,75]. In a retrospective study involving 338 patients undergoing Transcatheter Aortic Valve Replacement (TAVR) work-up, both coronary CTA and FFR_CT_ were compared to ICA as the reference for diagnosing obstructive CAD. FFR_CT_ significantly outperformed CCTA alone [76]. Specifically, per-patient sensitivity was 84.6% vs. 76.9%, specificity was 88.3% vs. 64.5%, and negative predictive value was 96.0% vs. 92.1%, respectively. FFR_CT_ demonstrated a higher diagnostic accuracy (87.6% vs. 66.9%) and AUC (0.90 vs. 0.84, *p* = 0.02). A key finding is that FFR_CT_ can increase the diagnostic yield of CCTA without additional procedures, reducing risks and contrast burden, particularly relevant in elderly and fragile patients where invasive testing carries increased procedural risk and where use of vasodilators may be contraindicated. Furthermore, an FFRCT-guided strategy would have safely avoided unnecessary ICA in 57.1% of patients compared to 43.6% when relying on CCTA alone. The integration of machine learning-based FFR_CT_ into routine coronary CTA interpretation for TAVI was further explored by Brandt et al. In a retrospective cohort of 95 patients, 12 were found to have functionally significant CAD based on invasive FFR [77]. While CAD Reporting and Data System (CAD-RADS) stratification alone, based solely on anatomical severity, missed 4 of these cases, the addition of FFR_CT_ (≤0.80) to patients with CAD-RADS 2–3 successfully identified all 12, achieving a sensitivity and negative predictive value of 100%. This approach would have safely reduced unnecessary invasive coronary angiography in 68% of the cohort. FFR_CT_ and CAD-RADS could successfully identify CAD prior to TAVR, improving risk stratification and therapeutic decision making.

Prospective feasibility of FFR_CT_ in the TAVI setting was confirmed in a single-center study, which evaluated 42 patients with severe aortic stenosis scheduled for both coronary CTA and invasive FFR prior to TAVI [78]. At the per-vessel level, the correlation between FFR_CT_ and invasive FFR was moderate (Pearson r = 0.64), with diagnostic accuracy of 76.7%, sensitivity 73.9%, specificity 78.4%, and AUC 0.83. Per-patient analysis yielded similar accuracy (76.9%), sensitivity (76.5%), specificity (77.3%), and AUC (0.81), supporting the technique’s clinical feasibility and reliability in this complex cohort. Wang et al. retrospectively assessed the diagnostic performance of CT-FFR_ML_ compared to ICA in 100 patients undergoing TAVI work-up [79]. At the per-patient level, CT-FFR_ML_ achieved a sensitivity of 86.4%, specificity of 66.1%, PPV of 66.7%, NPV of 86.0%, and overall accuracy of 75.0%. At the per-vessel level (n = 400), FFR_CT_ yielded an accuracy of 77.0%, sensitivity of 77.6%, specificity of 76.9%, PPV of 44.0%, and NPV of 93.6%, further validating the utility of CT-FFR_ML_ for pre-TAVI triage. Similarly, Göhmann et al. conducted a retrospective analysis of TAVI candidates to undergo CT-FFR_ML_ to rule out possible CAD [80]. CT-FFR_ML_ was performed in 216 patients who were positive for CAD in CCTA. Out of them, 17 were reclassified as CAD negative. The diagnostic accuracy was increased with the implementation of CT-FFR_ML_ by a Δ of 3.4%. The sensitivity of the method was 94.9%, the specificity was 52%, PPV 52.2%, and NPV 94.9%, making it an effective diagnostic tool in the reduction of unnecessary ICAs before TAVR. Unlike most studies evaluating FFR_CT_ as a non-invasive gatekeeper to ICA before TAVI, Zhang et al. uniquely investigated how TAVI alters coronary physiology over time by assessing serial changes in FFR_CT_ [81]. In 190 patients who underwent both baseline and one-year follow-up CCTA, the authors observed a significant improvement in FFR_CT_ among those with FFR_CT_ ≤ 0.80 pre-TAVI (mean increase from 0.763 to 0.822, *p* < 0.001), while patients with initially FFR_CT_ > 0.80 exhibited a small but statistically significant decline (from 0.8789 to 0.8718, *p* < 0.001). This study also identified the presence of CAD, LAD lesions, and higher stenosis severity as independent predictors of post-TAVI physiological deterioration. Despite these shifts, changes in FFR_CT_ were not associated with clinical events during follow-up.

### 4.6. In-Stent Restenosis (ISR)

The initial evidence for the clinical utility of FFR_CT_ in the assessment of in-stent restenosis (ISR) emerged from a case report by Andreini et al., who described a patient with multiple drug-eluting stents in whom severe ISR was missed by standard CCTA but was accurately identified by FFR_CT_ [82]. This case illustrates the incremental diagnostic value of FFR_CT_ over anatomical imaging in specific scenarios by showing a significant physiological drop distal to the right coronary artery stent (FFR_CT_ < 0.50), which was later confirmed by ICA and led to PCI.

A few years later, Tang et al. published the first study to validate the feasibility and prognostic value of machine learning–based FFR_CT_ in patients with prior stent implantation. In their two-cohort, retrospective analysis, per-patient accuracy of FFR_CT_ for detecting hemodynamically significant ISR (invasive FFR ≤ 0.88 as reference) was 0.85, with strong correlation to invasive FFR (ICC = 0.84). The prognostic value of serial CT-FFR_ML_ assessment was subsequently investigated in a cohort of 115 patients undergoing CCTA after PCI. Over a median follow-up of 25 months, 15.7% experienced MACE. Multivariable Cox analysis identified age (HR 1.10 per year; 95% CI: 1.032–1.177; *p* = 0.004) and follow-up ΔFFR_CT_/length (HR 1.014; 95% CI: 1.006–1.023; *p* = 0.001) as independent predictors of MACE, with a model c-index of 0.856. This study demonstrated that higher follow-up ΔFFR_CT_/length was associated with increased risk of adverse events, underscoring the method’s potential for functional risk stratification in stented segments.

## 5. Cost-Utility and Validation Evidence: A Critical Synthesis of FFR_CT_

Although substantial evidence indicates that FFR_CT_ offers superior diagnostic accuracy compared to CCTA alone, particularly for the identification of functionally significant ischemic CAD, important barriers such as cost, reimbursement, and workflow integration currently limit its universal adoption across all CCTA examinations. At present, the optimal use of FFR_CT_ is as an adjunctive problem-solving tool, employed selectively in cases where a coronary lesion of indeterminate functional significance is detected. For example, FFR_CT_ is especially valuable for the assessment of intermediate (30–70%) stenoses, heavily calcified or tandem lesions, and scenarios complicated by imaging artifact, where conventional anatomical assessment is inconclusive. Additionally, FFR_CT_ may also help guide decision-making in individuals, often younger and with low Atherosclerotic Cardiovascular Disease (ASCVD) risk scores, whose symptoms and traditional risk assessment fail to identify significant underlying disease [83].

Despite broadly similar diagnostic goals, current FFR_CT_ platforms differ significantly in their algorithmic foundations, computational logistics, regulatory status, and readiness for clinical integration. These differences carry direct implications for reliability, accessibility, and health-system adoption. HeartFlow FFR_CT_, which employs full-order CFD based on the Navier–Stokes equations, remains the most extensively validated platform. Key trials, including DISCOVER-FLOW, DeFACTO, NXT, and PACIFIC, have consistently demonstrated high diagnostic accuracy for the detection of functionally significant CAD when compared with invasive FFR. Furthermore, recent large studies have demonstrated that FFR_CT_-guided care is associated with clinical outcomes, including rates of MACE, death, MI, and revascularization, that are similar to standard functional or invasive strategies. In the PRECISE trial (n = 2103, median follow-up 11.8 months), a precision strategy of CCTA with selective FFR_CT_ reduced the rate of unnecessary catheterization without obstructive coronary disease compared with usual testing (2.6% vs. 10.2%, HR 0.24, 95% CI 0.16–0.36), with no significant difference in the composite of death or nonfatal MI (HR 1.52, 95% CI 0.73–3.15) [84]. The ADVANCE registry (n = 5083) showed low 1-year rates of MACE overall, and cardiovascular death or MI was significantly lower in patients with a negative FFR_CT_ (0.2%) compared to those with a positive FFR_CT_ (0.8%, RR 4.22, 95% CI 1.28–13.95, *p* = 0.01) [68]. In PLATFORM, the 90-day MACE rates were low and not statistically different between patients evaluated with a CCTA/FFR_CT_-guided strategy and those managed with usual care, while FFR_CT_ -guided care resulted in a substantial reduction in unnecessary ICA (12% vs. 73%, risk difference 61%, 95% CI 53–69, *p* < 0.0001) [43]. It should be noted that these studies were conducted in structured research settings with high image quality and specialized referral pathways, which may not fully represent outcomes in routine practice.

In contrast, reduced-order models such as cFFR_ML_ offer dramatically shorter computation time, down to an execution time of approximately 2.4 ± 0.44 s, by utilizing deep learning networks trained on a large database of algorithmically generated (synthetic) coronary anatomies, with reference FFR values computed using a validated reduced-order CFD model [22]. This innovation allows seamless on-site deployment and faster integration into emergency or outpatient workflows. However, these platforms lack large-scale clinical outcome data and have not been subjected to formal economic modeling or cost-utility analyses. DeepVessel FFR, an AI-native platform, enables rapid computation by employing a dual-architecture deep learning model that automates coronary artery segmentation and FFR prediction directly from CCTA images, removing the need for manual preprocessing. Despite favorable diagnostic accuracy and widespread regulatory clearance (FDA, CE, NMPA), it similarly lacks prospective outcome studies and economic evaluations. The variability in modeling assumptions, such as Newtonian versus non-Newtonian blood flow, steady-state versus transient simulation, and differences in boundary condition estimation, can lead to discrepancies in physiological estimates across platforms, particularly in cases of complex coronary anatomy or high calcium burden. Regulatory heterogeneity further complicates adoption; HeartFlow is FDA-cleared, CE-marked, and NICE-endorsed, while DVFFR has received FDA and CE clearance as well as regulatory approval in China and Singapore. By contrast, most other platforms are still under research validation without formal market clearance.

Recent economic analyses have offered perspective on the economic and clinical impact of FFR_CT_-guided diagnostic strategies. For example, in a contemporary tertiary care cohort, Rasoul et al. found that the adoption of FFR_CT_ did not lead to overall cost savings [85]. In fact, average per-patient costs were slightly higher with FFR_CT_-guided care than with direct-to-invasive angiography strategies, especially in settings where the prevalence of obstructive coronary disease was high and the ability to avoid unnecessary ICA was limited. Similarly, Graby et al. reported from the UK National Health Service that, although FFR_CT_-based strategies led to changes in clinical management, such as the cancellation of functional imaging in 17% and invasive angiography in 47% of cases, the net increase in per-patient cost was marginal [86]. The authors concluded that the clinical and economic benefits of FFR_CT_ are most evident when patient selection aligns with guideline-based criteria. Specifically, reserving FFR_CT_ for cases with CCTA-detected stenosis ≥50%, while broader use in less-selected populations may increase costs without additional clinical value. Furthermore, a thorough individual-based Markov microsimulation by Karády et al., using PROMISE trial data, showed that CCTA with selective FFR_CT_ was cost-effective, often even economically dominant, compared to functional testing for low-risk stable chest pain, provided test costs and patient selection remained appropriate [87]. However, sensitivity analyses demonstrated that the cost-effectiveness of anatomic strategies was maintained across variations in statin adherence and expanded use of FFR_CT_, with probabilistic analyses showing that CCTA and CCTA + FFR_CT_ were cost-effective compared to functional testing in 69.4% and 65.4% of scenarios, respectively, at a willingness-to-pay threshold of $100,000 per quality-adjusted life-year (QALY). In contrast, a large real-world audit from the UK NHS found that FFR_CT_ was used according to NICE criteria in only 40% of cases, and that, in clinical practice, FFR_CT_-guided pathways were associated with both lower diagnostic accuracy and higher per-patient cost (£2102 for FFR_CT_ vs. £1242–1580 for stress imaging). This analysis suggested that indiscriminate use of FFR_CT_, outside of tightly defined clinical indications, can diminish both clinical and economic value [88].

By contrast, the PLATFORM trial, conducted across multiple European centers, remains the most thorough controlled assessment of HeartFlow FFR_CT_’s clinical and economic implications [43]. They evaluated a CCTA with a selective FFR_CT_ strategy versus usual care in patients with stable chest pain. Among patients with a planned invasive coronary angiogram, the FFR_CT_-guided strategy resulted in a 61% cancellation rate of planned ICAs, and a significant reduction in unnecessary ICAs for non-obstructive disease: 12.4% in the FFR_CT_ arm versus 73.3% with usual care (risk difference 60.8%, 95% CI 53.0–68.7%, *p* < 0.0001) at 90 days. At 1-year follow-up, clinical outcomes and quality of life were similar between groups, while mean cumulative costs were significantly lower in the FFR_CT_ group compared to usual care ($8127 vs. $12,145; difference –$4018, 95% CI $1590 to $6577; *p* < 0.0001), representing a 33% cost reduction [44]. Additionally, in a pre-specified analysis of the PLATFORM trial, FFR_CT_-guided care for patients referred for noninvasive testing led to significantly greater improvements in the quality of life compared to usual care, as measured by the Seattle Angina Questionnaire (mean change 19.5 vs. 11.4, *p* = 0.003) and EuroQOL scores (0.08 vs. 0.03, *p* = 0.002). In the invasive testing stratum, this approach reduced unnecessary invasive procedures and lowered mean 90-day costs by 32% ($7343 vs. $10,734, *p* < 0.0001) [89].

Despite these encouraging results, HeartFlow’s per-analysis cost, approximately $997 in the U.S, remains a barrier to widespread adoption, particularly where reimbursement is not well established [90]. Moreover, the technology’s dependence on off-site processing and high-quality CCTA limits accessibility in resource-constrained settings. While newer platforms such as cFFR_ML_, DVFFR, uFFR_CT_, and esFFR_CT_ promise faster processing and on-site analysis, as of 2025, there are no published economic data or prospective multicenter outcome studies for these alternatives. Although these methods have shown promising diagnostic accuracy in smaller or retrospective cohorts, their comparative clinical and economic value remains unproven, and any extrapolation of HeartFlow data to these technologies should be made with caution. Most AI-based FFR_CT_ techniques remain investigational, and real-world clinical implementation, including reimbursement and workflow optimization, requires further study. Thus, methodological heterogeneity, differences in validation rigor, and the absence of economic analyses reinforce the need for multi-vendor, prospective head-to-head trials to inform an evidence-based integration of FFR_CT_ into a variety of clinical settings.

## 6. Proposed Algorithm for FFR_CT_ Integration in Clinical Practice

**Figure 1:**
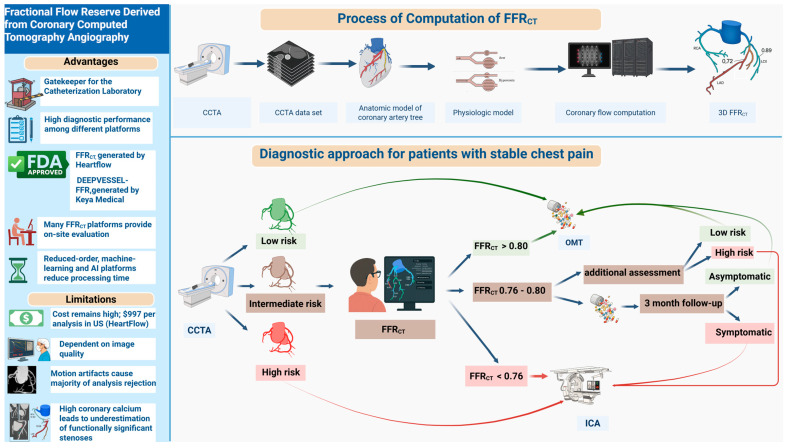
Computation pipeline, advantages, limitations, and diagnostic approach of coronary computed tomography-derived fractional flow reserve (FFR_CT_) in stable chest pain. Created in BioRender. Bozika, M. (2025) https://BioRender.com/dcmmr1g, accessed on 10 July 2025.

The integration of FFR_CT_ into the diagnostic pathway is primarily recommended for patients with intermediate anatomical risk, where anatomical stenosis on CCTA does not adequately predict functional significance (Figure 1). The term intermediate-risk anatomy is defined as a 30–49% luminal stenosis in the left main, 30–69% in the left anterior descending artery (LAD), and ≥70% stenosis in other epicardial coronary vessels such as the right coronary artery (RCA) and left circumflex artery (LCx). High-risk anatomy includes ≥50% left main stem stenosis, ≥70% LAD stenosis, and three-vessel disease or complete occlusion. Low-risk anatomy is characterized by stenoses less than these thresholds [91,92].

The use of FFR_CT_ in patients with intermediate-risk stenosis can identify lesion-specific ischemia and guide downstream decision-making, including the avoidance of unnecessary ICA. In case of high-risk stenosis, direct referral to ICA and potential revascularization is appropriate, whereas in low-risk patients, OMT and clinical surveillance are typically recommended. A proposed diagnostic algorithm recommends performing FFR_CT_ when CCTA identifies intermediate-risk lesions, while reserving invasive strategies for those with high-risk features and conservative management for those with low-risk profiles.

The figure illustrates the full workflow and clinical integration of FFR_CT_:

(A) Advantages and Limitations: The left panel summarizes the principal advantages of FFR_CT_, including its role as a non-invasive “gatekeeper” to invasive angiography, high diagnostic performance across platforms, U.S. Food and Drug Administration (FDA) approval, on-site evaluation availability, and the potential for rapid processing via reduced-order, machine learning-based, or AI-based approaches. Key limitations include high per-analysis cost, dependency on image quality, frequent analysis rejection due to motion artifacts, and reduced accuracy in the setting of high coronary calcium.
(B) Process of Computation: The upper panel depicts the FFR_CT_ computational pipeline: acquisition of coronary computed tomography angiography (CCTA) data, 3D reconstruction of the coronary artery tree, development of a physiologic model, and computational simulation of coronary flow to yield vessel- and lesion-specific FFR_CT_ values.
(C) Diagnostic Approach: The lower panel demonstrates a contemporary diagnostic algorithm for patients presenting with stable chest pain. Following CCTA-based anatomical risk stratification (low, intermediate, high), patients at intermediate risk undergo FFR_CT_ analysis. Management is then tailored according to FFRCT values, as follows:
  - FFR_CT_ > 0.80: Low risk, optimal medical therapy (OMT) recommended.
  - FFR_CT_: 0.76–0.80: Further clinical assessment and short-term follow-up
  - FFR_CT_ < 0.76: Referral for invasive coronary angiography (ICA).

Pathways diverge further based on patient symptoms and follow-up findings.

## 7. Limitations

Despite its demonstrated value, FFR_CT_ has several notable limitations. The diagnostic performance of FFR_CT_ is highly dependent on image quality, which can be compromised by motion artefacts, intracoronary stents, cardiac devices, prosthetic valves, or extensive coronary calcification [18,23,29,93,94,95]. In clinical trials such as NXT [29] and PLATFORM [43], approximately 10–12% of datasets were rejected for FFR_CT_ analysis due to suboptimal imaging. Extensive coronary calcification significantly impairs the diagnostic performance of FFR_CT_ [96,97]. Among patients with false negative FFR_CT_ results, the majority exhibited high calcium scores (622–1108 HU), which indicates that heavily calcified plaques may distort pressure-flow estimates and lead to an underestimation of functionally significant stenoses [85]. Invasive evaluation or alternative functional imaging in this subgroup should be considered [85]. Furthermore, motion artifacts are the leading cause of FFR_CT_ rejection, accounting for up to 78% of failed cases in the ADVANCE registry and 64% in routine clinical practice. These artifacts, typically resulting from suboptimal cardiac motion control or patient cooperation, impair the reconstruction of accurate coronary models. Strict adherence to acquisition protocols, including heart rate control and image quality optimization, is important to enable reliable physiological assessment [98].

Another important limitation of FFR_CT_ technology, particularly with CFD-based platforms such as HeartFlow, is the prolonged image processing time, which frequently requires several hours. Although machine learning–based solutions offer the promise of rapid, on-site FFR_CT_ computation, these platforms are still undergoing validation and have yet to achieve widespread clinical implementation [91]. As of 2024, HeartFlow remains the only FFR_CT_ platform with FDA clearance. Another barrier is the procedural cost, where HeartFlow’s list price in the United States is approximately $997 per analysis. However, economic data from the PLATFORM trial and subsequent studies suggest that the use of FFR_CT_ may yield overall cost savings by reducing unnecessary angiography and revascularization [90].

## 8. Future Directions

A major future challenge and opportunity for CCTA and FFR_CT_ lies in the optimal integration of AI, not as a replacement for the expertise of cardiologists, but as a supportive tool to enhance reporting accuracy, workflow efficiency, and individualized prognostic assessment. Human interpretation, despite the experience of expert readers, remains susceptible to fatigue and inherent variability, while the training of such specialists requires years of dedicated practice. The anticipated growth in CCTA and FFR_CT_ demand, driven by expanding guideline recommendations, further amplifies the need for efficient tools that can reduce interpretation time and facilitate the delivery of actionable information.

Importantly, the next phase of FFR_CT_ interpretation is shifting toward a precision medicine paradigm. Comprehensive reporting should incorporate plaque characterization, assessment of hemodynamic impact, and integration of clinical parameters to enable detailed risk stratification and individualized treatment planning. AI algorithms, particularly those capable of integrating imaging with clinical data, hold promise to support this more sophisticated approach.

However, various barriers must be addressed before AI-based technologies may be adopted in healthcare settings. Regulatory pathways vary by jurisdiction: in the United States, AI systems designed to assist in quantitative image analysis may be eligible for FDA 510(k) clearance, whereas those designed for autonomous clinical interpretation require pre-market approval and comprehensive evidence of safety and accuracy from clinical trials. In Europe, medical device and data protection legislation already provides significant safeguards, but new regulations are being established to assure the openness and human oversight of clinical AI applications [99].

From a technical perspective, two major approaches are being developed to solve generalizability and privacy problems. The first approach uses transfer learning, in which algorithms trained on local data are modified in new populations to improve performance. This is especially useful when data exchange is limited owing to privacy concerns or demographic differences. The second approach entails working together to build huge, high-quality open-access datasets, which will make it easier to develop and assess more accurate, widely applicable algorithms [99].

Finally, true clinical integration of AI will necessitate not only technological and legal breakthroughs but also thorough validation in real-world practice, proof of improvements in efficiency in clinical workflow, and assurance of equality and cybersecurity across all applications [100]. The medical and AI groups must work closely together to improve regulatory frameworks, develop realistic standards for implementation, and address potential ethical concerns such as bias and inequities in access.

## 9. Conclusions

In clinical practice, our synthesis of recent evidence indicates that FFR_CT_ should be considered as an adjunctive tool in the diagnostic assessment of patients with suspected CAD, particularly when CCTA identifies intermediate (30–70%) stenoses, heavily calcified lesions, or cases where image artifacts limit the reliability of anatomical assessment. By integrating lesion-specific functional information into the decision-making process, FFR_CT_ can help clinicians more confidently select patients who truly require invasive coronary angiography or revascularization, while safely deferring intervention in those with hemodynamically insignificant disease. This targeted approach has the potential to reduce unnecessary invasive procedures, optimize resource utilization, and minimize patient risk.

However, successful adoption of FFR_CT_ in routine care depends on practical considerations, including access to the technology, processing time, cost, and local reimbursement policies. HeartFlow FFR_CT_, with the strongest validation in prospective studies, is well suited for use as a problem-solving tool in patients with intermediate, while newer AI-based and ML-based platforms offer the promise of faster turnaround but require further real-world validation. Importantly, our review underscores that indiscriminate use of FFR_CT_ outside guideline-based indications may increase costs without clear clinical benefit. Therefore, clinicians should employ FFR_CT_ selectively, guided by both patient characteristics and institutional resources, and remain attentive to novel evidence from multi-vendor, head-to-head trials. As the field evolves, integrating FFR_CT_ thoughtfully into the diagnostic pathway could lead to more accurate, effective, and patient-centered treatment.

## Figures and Tables

**Table 1:** Guideline recommendations for coronary computed tomography angiography and FFR_CT_ across common clinical scenarios. Comparison of SCCT 2021 Expert Consensus and 2021 Multisociety Guidelines.

Clinical Scenario	SCCT 2021 Expert Consensus	2021 AHA/ACC/ASE/Chest/SAEM/SCCT/SCMR Multisociety Guidelines
Stable chest pain (no known CAD)	First-line test in low- to intermediate-risk patients	Class I (Level of Evidence A): Recommended when intermediate stenosis present; improves lesion-specific assessment
Acute chest pain (low- to intermediate-risk)	Supports ECG—gated CCTA in emergency setting for early rule-out, including the “triple rule-out” approach (CAD, pulmonary embolism, aortic dissection) in appropriately selected patients (men > 45, women > 55).	Acute chest pain (low- to intermediate-risk)
Inconclusive CCTA	Supports use of additional non-invasive imaging (FFR_CT_, CTP, or alternative modalities such as CMR, nuclear perfusion imaging, or stress echocardiography); selection among these non-invasive functional tests is limited.	Class IIa (Level of Evidence B): Recommends downstream non-invasive testing: FFR_CT_ or myocardial perfusion imaging, and notes that the choice should be individualized based on test availability, patient-specific contraindications (e.g., MRI compatibility); suggesting a shared decision-making approach.
Intermediate stenosis (30–90% diameter stenosis)	Recommends functional assessment with FFR_CT_ or CTP to clarify ischemic significance	Class IIa (Level of Evidence B): Supports downstream testing (FFRCT or MPI); acknowledges improved diagnostic accuracy when paired with CCTA
Asymptomatic high-risk individuals (diabetes, smoking, inflammatory conditions, familial hypercholesterolemia, HIV, or a strong family history)	Conditionally appropriate for detecting subclinical CAD and initiating preventive therapies	Not routinely recommended, but may be considered in selected high-risk patients (e.g., diabetes, strong family history, chronic inflammatory conditions)
Asymptomatic low- or intermediate-risk individuals	Rarely appropriate due to low yield and risk of downstream harm	Not recommended due to low diagnostic yield
Prior CABG	Recommended for graft patency evaluation; native vessel assessment limited by artifacts	Class IIb (Level of Evidence B): Recommended for evaluating graft patency
Stents ≥ 3 mm	Appropriate for ISR evaluation	N/A
Congenital coronary anomalies	Recommended as first-line due its ability to accurately depict complex three-dimensional anatomical relationships, vessel courses, and potential high-risk features such as interarterial or intramural courses.	N/A
New cardiomyopathy	Supports use of CCTA to identify ischemic etiology in unexplained cardiomyopathy; Appropriate to exclude obstructive coronary artery disease	N/A
Heart transplant (coronary allograft vasculopathy surveillance)	Recognized as non-invasive alternative to invasive angiography in transplant recipients, when renal function allows contrast use	N/A
Viability/scar assessment (when MRI contraindicated)	Not guideline-emphasized; considered investigational or conditional in MRI-limited patients Delayed-enhancement CCTA is an acceptable alternative in specific revascularization or ablation planning settings	N/A

Abbreviations: CAD: Coronary Artery Disease, ECG: Electrocardiogram, CCTA: Coronary Computed Tomography Angiography, FFR_CT_: CT-derived Fractional Flow Reserve, CTP: CT-myocardial perfusion imaging, CMR: Cardiac Magnetic Resonance, MPI: Myocardial Perfusion Imaging, HIV: Human Immunodeficiency Virus, ISR: In-Stent Restenosis, CABG: Coronary Artery Bypass Graft Surgery, N/A: Not Available, MRI: Magnetic Resonance Imaging.

**Table 2:** ^†^ Major studies investigating the diagnostic performance of currently available Fractional Flow Reserve Derived from Coronary Computed Tomography Angiography (FFR_CT_) techniques, presented in chronological order according to the introduction of each technique.

3D Software Provider	Study	Parameter	Patients (n)	Vessels (n)	Design	AUC	Accuracy (%)	Sensitivity (%)	Specificity (%)	PPV (%)	NPV (%)	Correlation Coefficient
Heartflow	DISCOVER FLOW 2011 [23]	FFR_CT_	103	159	Prospective, multicenter	0.90	84.3	87.9	82.2	73.9	92.2	0.678
Heartflow	DeFACTO 2013 [28]	FFR_CT_	252	407	Prospective, multicenter	0.79	69	74	67	41	90	0.50
Heartflow	HTNXT 2014 [29]	FFR_CT_	254	484	Prospective, multicenter	0.93	86	84	86	61	95	0.82
Heartflow	Yang et al. 2016 [30]	FFR_CT_	72	138	Retrospective	0.919	81	87	77	71	90	0.671
Heartflow	Driesen et al. 2019 [31]	FFR_CT_	157	505	Post-hoc analysis of PACIFIC trial	0.94	87	90	86	65	96	0.80
Siemens (1st generation)	Renker et al. 2014 [32]	cFFR	53	67	Retrospective	0.92	N/A	85	85	71	93	0.66
Siemens (1st generation)	Coenen et al. 2015 [21]	cFFR	106	189	Retrospective	0.83	74.6	87.5	65.1	64.8	87.7	0.59
Siemens (1st generation)	De Geer et al. 2016 [33]	cFFR	21	23	Retrospective	N/A	78	83	76	56	93	0.77
Siemens (2nd generation)	Itu et al. 2016 [22]	cFFR_ML_	87	125	Prospective, single center	N/A	83.2	81.6	83.9	68.9	91.2	0.729
Toshiba	Ko et al. 2017 [34]	FFR_CT_	30	56	Prospective, single center	0.88	83.9	77.8	86.8	73.7	89.2	0.57
Keya Medical Technology	Wang et al. 2019 [35]	DEEPVESSEL-FFR	63	71	Prospective, single center	0.933	88.73	97.56	76.67	85.11	95.83	0.683
Keya Medical Technology	ADAPT 2021 [36]	DEEPVESSEL-FFR	302	N/A	Retrospective, multicenter	N/A	86.8	86.9	86.7	79.4	91.9	N/A
United-Imaging Healthcare	Tang 2020 [38]	uCT-FFR	338	422	Retrospective, multicenter	0.92	91	89	91	86	94	0.69
CAscope	Ding 2023 [39]	esFFR	329	350	Prospective, multicenter	0.97	93	95	92	90	96	0.81

^†^ Diagnostic performance is reported on a per-vessel or per-lesion basis. FFR_CT_: Fractional Flow Reserve Derived from Coronary Computed Tomography Angiography; cFFR: coronary computed tomography angiography–derived computational FFR; cFFR_ML_: machine-learning-based fractional flow reserve from coronary computed tomography; uCT-FFR: computational fluid dynamics–based CT FFR; esFFR: coronary flow dynamics-based CT-derived FFR algorithm; N/A: Not Available.
